# Prognostic stratification of adult primary glioblastoma multiforme patients based on their tumor gene amplification profiles

**DOI:** 10.18632/oncotarget.25562

**Published:** 2018-06-15

**Authors:** María González-Tablas, Inês Crespo, Ana Luísa Vital, Álvaro Otero, Ana Belén Nieto, Pablo Sousa, María Carmen Patino-Alonso, Luis Antonio Corchete, Hermínio Tão, Olinda Rebelo, Marcos Barbosa, Maria Rosário Almeida, Ana Filipa Guedes, María Celeste Lopes, Pim J. French, Alberto Orfao, María Dolores Tabernero

**Affiliations:** ^1^ Centre for Cancer Research (CIC IBMCC-CSIC/USAL), Department of Medicine, CIBERONC, University of Salamanca, Salamanca, Spain; ^2^ Centre for Neuroscience and Cell Biology, University of Coimbra, Coimbra, Portugal; ^3^ Faculty of Pharmacy, University of Coimbra, Coimbra, Portugal; ^4^ Servicio de Neurocirugía, Hospital Universitario e Instituto Biosanitario de Salamanca (IBSAL), Salamanca, Spain; ^5^ Department of Statistics, University of Salamanca, Salamanca, Spain; ^6^ Departamento de Hematología, Hospital Universitario, IBSAL, IBMCC (USAL-CSIC), Salamanca, Spain; ^7^ Neurosurgery Service, University Hospital of Coimbra, Coimbra, Portugal; ^8^ Neuropathology Laboratory, Neurology Service, University Hospital of Coimbra, Coimbra, Portugal; ^9^ Faculty of Medicine, University of Coimbra, Coimbra, Portugal; ^10^ Department of Neurology, Erasmus MC, Rotterdam, The Netherlands; ^11^ Instituto Biosanitario de Salamanca (IBSAL), Salamanca, Spain

**Keywords:** glioblastoma, classification, subtypes, gene amplification, survival

## Abstract

Several classification systems have been proposed to address genomic heterogeneity of glioblastoma multiforme, but they either showed limited prognostic value and/or are difficult to implement in routine diagnostics. Here we propose a prognostic stratification model for these primary tumors based on tumor gene amplification profiles, that might be easily implemented in routine diagnostics, and potentially improve the patients management. Gene amplification profiles were prospectively evaluated in 80 primary glioblastoma multiforme tumors using single-nucleotide polymorphism arrays and the results obtained validated in publicly available data from 267/347 cases. Gene amplification was detected in 45% of patients, and chromosome 7p11.2 including the *EGFR* gene, was the most frequently amplified chromosomal region – either alone (18%) or in combination with amplification of DNA sequences in other chromosomal regions (10% of cases). Other frequently amplified DNA sequences included regions in chromosomes 12q(10%), 4q12(7%) and 1q32.1(4%). Based on their gene amplification profiles, glioblastomas were subdivided into: i) tumors with no gene amplification (55%); ii) tumors with chromosome 7p/*EGFR* gene amplification (with or without amplification of other chromosomal regions) (38%); and iii) glioblastoma multiforme with a single (11%) or multiple (6%) amplified DNA sequences in chromosomal regions other than chromosome 7p. From the prognostic point of view, these amplification profiles showed a significant impact on overall survival of glioblastoma multiforme patients (p>0.001). Based on these gene amplification profiles, a risk-stratification scoring system was built for prognostic stratification of glioblastoma which might be easily implemented in routine diagnostics, and potentially contribute to improved patient management.

## INTRODUCTION

Primary glioblastoma multiforme (GBM) is the most common and malignant subtype of glial tumors [1]. From the clinical and biological point of view, GBM includes a rather heterogeneous group of tumors that vary by site of origin, histophatological features, tumor microenvironment [2] and genetics [3]. They are usually resistant to radio/chemotherapy and show overall survival (OS) rates of a few months to years, making them unvariably lethal [4, 5]. Criteria used for the histological classification and grading of GBM have been recently revised in the 4^th^ World Health Organization (WHO) classification of GBM [1]; however, this classification still fails in distinguishing subgroups (or variants) of primary GBM that display clearly distinct clinical and biological behaviours, despite sharing similar histopathological features [6–9].

Besides histopathology, molecular genetics data has also confirmed the high heterogeneity of GBM, both at the intertumoral and intratumoral levels [3, 10–12]; in addition, molecular genetics data also proved useful for improving the diagnosis, classification, and prognostic stratification of GBM [13, 14], with therapeutic implications [14–16]. However, despite all advances achieved via the study of the methylation status, gene mutations (e.g. *IDH1 or PT53* genes) [17] and affected oncogenic pathways [11, 18–20], the precise mechanisms involved in the pathogenesis of GBM still remain far from being fully understood. In turn, routine implementation of molecular genetics into the diagnostic classification of GBM still remains limited, due to the complexity of the genetic findings involved.

Among other genetic abnormalities, the copy number aberration (CNA) profile of tumor cells, over the copy number variation (CNV) of individual patients, has been recognized [21–24] as a useful prognostic tool in GBM [14]. Thus, some copy number aberrations involving one or multiple genes that affect a significant fraction of the tumors, (e.g. CNA associated with either gains of chromosome 7 and/or amplification of the epidermal growth factor receptor (*EGFR*) gene [18], and losses of DNA sequences in chromosome 10 [19], together with other less frequent alterations involving DNA sequences in chromosomes 12q13-15 [25], 4q and 1q [26], have all been associated with the outcome of GBM patients. Despite this, risk stratification of GBM into classical and non-classical GBM, based on gene expression profiling (GEP) data, was first proposed [27]. Later on, Phillips et al [13] defined three molecular subtypes of GBM according to a combination of GEP data and numerical alterations of chromosomes 7 and 10: proneural, proliferative and mesenchymal GBM. This classification was subsequently redefined by the Cancer Genome Atlas Consortium (TCGA) [14] into four subtypes – proneural, proliferative, mesenchymal and neural GBM, using a combination of GEP data and CNA, together with the pattern of somatic mutations. Despite overlapping data is used in the later two classifications, both approaches are not equivalent, at the same time they are rather complex to be reproducibly applied in routine laboratory diagnostics. In addition, the prognostic impact of both approaches remains controversial because e.g. the proneural subgroup of Philips [13] has a longer survival, while proneural tumors defined according to the TCGA classification [14], have a poorer outcome. In turn, despite *EGFR* amplification is a defining event for the classical subtype of GBM, in the TCGA classification it appears in >95% of the neural and mesenchymal tumor subtypes, but also in 54% of the proneural subtype; similarly, the same *EGFR* mutations (e.g. the *EGFRvIII* variant) can also be detected across all above subtypes of GBM; in addition, neither the criteria used to define the proneural class based on focal amplification of the 4q12 locus harboring the *PDGFRA* gene (with or without *EGFR* amplification), nor the evaluation of the expression of the *NEFL, GABRA1, SYT1* and *SLC12A5* genes, are currently applied for routine diagnostic classification of GBM. Altogether this hampers fast and reproducible risk-stratification of GBM patients at diagnosis, based on this classification.

Here we investigated the gene amplification profiles (GAP) across the whole tumor genome of a series of 80 GBM tumors, as detected by high-density single-nucleotide polymorphism (SNP)-arrays, and evaluated their impact on overall survival (OS) of GBM patients. Based on the results obtained, a GAP-based risk-stratification model was built and validated in a series of 267 GBM tumors from a total of 7 GBM series publicly available at the GEO and ArrayExpress databases and/or whose data was kindly provided by the authors [15, 24, 28–32], in addition to our own cases.

## RESULTS

### Gene amplification profiles and chromosomal regions involved in GBM

**Figure 1:**
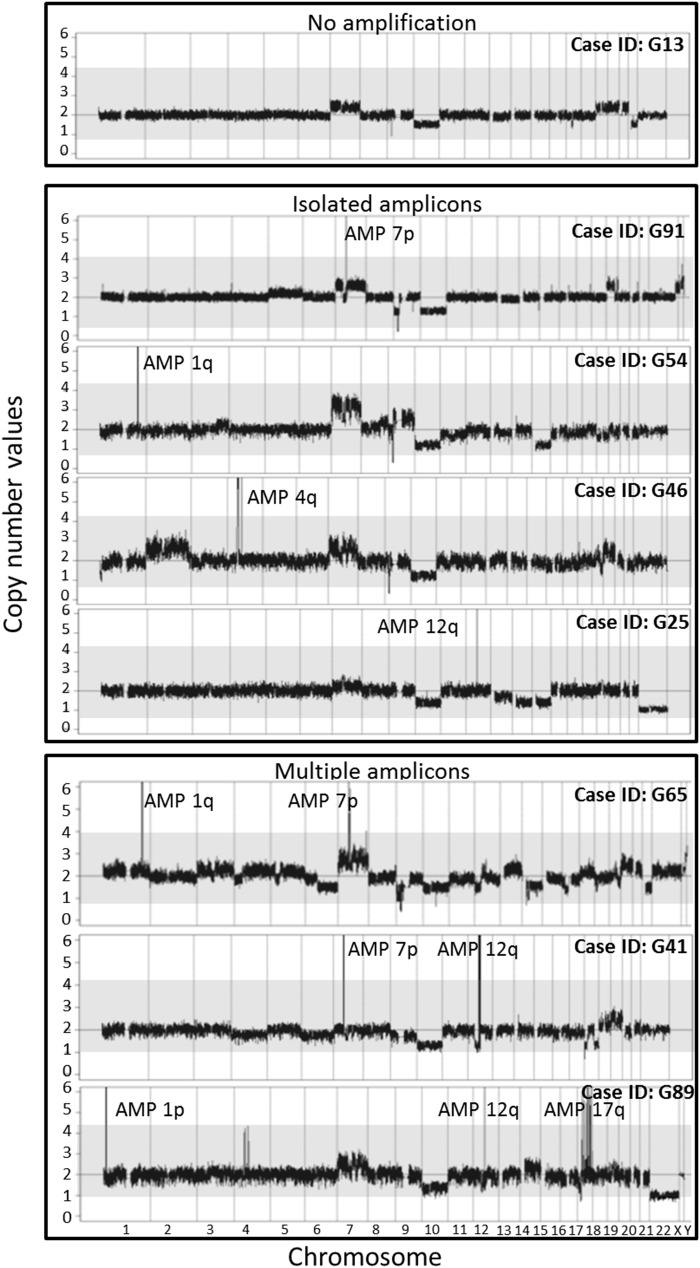
Illustrating examples of the cytogenetic profiles representative of the distinct patterns of gene amplification identified in GBM tumors from series 1, used for the definition of the 5 different subgroups of GBM based on their gene amplification profiles

SNP-arrays showed CNA containing > 7 DNA copies of the same DNA sequence for ≥1 chromosome/chromosomal region in all (80/80) cases analyzed from series 1. In the majority of patients (45/80; 56%) such CNA involved genetic amplification of DNA sequences from ≥1 chromosomal region (Table 1 and Figure 1). As expected, DNA sequences at chromosome 7p11.2 containing the *EGFR* gene, were the most frequently amplified sequences (30/80 cases; 38%), followed by DNA sequences at the 12q (14/80; (18%), 4q (8/80; 10%) and 1q (5/80; 6%) chromosomal regions.

**Table 1:** Major subsets of GBM that carried different gene amplification profiles, as identified in our series (series 1) of GBM patients (n=80) grouped according to the location, type and number of chromosomal regions involved (n=45/80 tumors)

Tumor group		Tumor ID	Amplified chromosome(s)	N. of amplified chromosomal band(s)/genes	Amplified Chromosomal bands
Gene amplification at a single chromosomal region (n=27)	*EGFR gene* involved (n=17;21%)	G94 G55 G91 G80 1/3G72 G68 G67 G56 G44^ϑ^ G40^ϑ^ G37^ϑ^ G30^ϑ^ GBM3^ϑ^ GBM7^ϑ^ GBM11^ϑ^ GBM12^ϑ^ GBM17^ϑ^	7p	2/4 2/2 1/2 1/2 1/2 1/1 1/2 1/3 1/3 1/2 1/1 1/17 1/3 1/7 1/25 1/2	7p11.2/7p12.1^#^ 7p11.2
	*EGFR gene* not involved (n=10;13%)	G73 G12 GBM1^ϑ^ GBM14^ϑ^ G51^ϑ^ G46^ϑ^ G25^ϑ^ G79 G54 G10	4q 12q 1q 16q#	3/35 1/29 1/16 1/7 2/14 2/5 1/8 1/12 1/18 1/0	4q11/4q12/4q13.3 4q11^#^/4q12 4q12 4q12 12q13.3/12q14.1 12q14.1-q14.3/12q15 12q14.1 1q32.1 16q12.1-q12.2
Gene amplification at multiple chromosomal regions (n=18)	*EGFR* gene & other amplicons involved (n=13;16%) *EGFR gene* not involved (n=5;6%)	G39^ϑ^ G41 G53^ϑ^ G70 G83 G65 GBM19^ϑ^ G23^ϑ^ GBM4^ϑ^ G82 G90 G81 G71 GBM13^ϑ^ GBM22^ϑ^ G08^ϑ^ G88 G89	7p, 12q 1q, 7p 7p, 7q 7p, 11p 1q,7p,12q 4q, 7p, 12q 5q^#^, 6q^#^, 7p 7p, 17p, 17q 7p, 11p, 11q, 12q 4q, 12q 1p, 7q 4q, 7q, 12q 1p, 12q, 17q	1/2, 2/9 1/4, 2/11 3/6, 1/2 1/1, 2/20 1/16, 1/1 1/26, 1/12 4/5 1/2, 1/17 1/18, 1/6, 2/11 2/6, 1/1, 3/23 1/1 1/2, 2/11 2/5, 3/16, 2/19 1/7, 1/11 1/19, 2/20 3/30, 1/7 2/13, 1/3, 4/30 1/3, 2/21, 3/6	7p11.2/12q13.3/12q14.1 7p11.2/12q14/12q15 7p11.2/7p21.3/7p22.1/12q15 7p11.2/12q13.3/12q14.1 1q32.1/7p11.2 7p21/7p12.3/7p11.2/7q22.3 7p11.2/11p13 1q32.1/7p11.2/12q13.3/12q14.1 4q12/4q13.3/7p11.2/12q13.12- q13.13/12q13.3/12q14.1/12q15 5q34/6q25/7p11.2 7p11.2/17p13.1/17q25.1 7p11.2/7p12.1/11p15.3/11p11.2^#^/ 11q13.3/11q25/12q13.3/12q14.1 4q12/12q14.1 4q12/12q13.3/12q14.1 1p12/1p13/1p21/7q21.2-q21.3 4q12/4q13.3/7q31.2/12q13.3/12q14.1/12q15/12q21.1 1p36.21/12q13.3/12q14.1/17q11/17q12^#^/17q21/17q22^#^/17q24

ID: Case identification code. Most frequently involved genes per chromosomal band included: 1q32.1, *MDM4*; 4q12, *PDGFRA*; 12q14.1, *CDK4*; 12q15, *MDM2*; 7q31.2,*MET*. ^#^: Amplified chromosomal region without any annotated gene in it; ^ϑ^: EGFR gene mutation studied in this sample.

Amplification of DNA sequences at chromosome 7p11.2 was found either alone (17/80; 21%) or in combination with amplification of DNA sequences at other regions in chromosomes 1q, 4q, 5q, 6q, 11p, 11q, 12q, 17p and 17q (13/80; 16% of cases) (Table 1). The most frequent combination of simultaneously amplified DNA sequences at chromosomal regions involving 7p11.2 were those of 7p and 12q (7/80 cases; 9%) and of 7p and 11p (2/80 cases; 3%) (Table 1).

Isolated amplification of DNA sequences at chromosomal regions other than 7p11.2 was found in 10/80 cases (13%); this included isolated amplification of 4q12 (where the *PDGFRA* gene is coded) in 4/80 cases (5%), 12q14.1 (where the *CDK4* gene is coded) in 3/80 cases (4%), 1q32.1 (this chromosomal region contains the *MDM4* gene) in another 2/80 tumors (3%) and 16q in one tumor (1%) without any annotated gene being coded in the amplified DNA sequences from this later chromosomal region (Table 1).

In turn, genetic amplification at 12q was detected in 14/80 cases (18%) including isolated amplification in 3 GBM and combined 12q gene amplification with gene amplification at other chromosome regions (1p, 1q, 4q, 11p, 11q, 7q, or 17q) in 11 tumors (Table 1), including amplification at chromosome 7p in 7 cases (9%). Gene amplification at 4q was found in 8/80 cases (10%) either alone (4/80; 5% or in association with amplification of DNA sequences at the 12q and/or 7p (4/80; 5%) chromosomal regions (Table 1).

Overall, coexistence of genetically amplified DNA sequences at ≥ 2 chromosomal regions in the same tumor (median of 2 altered chromosomal regions; range: 2-4 regions) was found in 18 cases (23%). In 13 of these 18 tumors, the *EGFR* gene was involved (Table 1). Of note, gene amplification involving regions at chromosomes 7q (4%), 11p (3%), 17q (3%), 5q (1%), 6q (1%), 11q (1%) and 17p (1%) included variable chromosomal patterns and numbers of genes involved (Table 1 and Table 2). Although gene amplification was frequently associated with chromosomal gains (polyploidy), there were also cases with polyploidies in the absence of gene amplification and vice versa.

**Table 2:** Frequency of genetic amplification involving different chromosomal regions in the test (series 1) vs the validation series (series 2-8) of GBM patients analyzed

Number of amplified regions	Chromosomal regions involved	Amplified GBM cases	
		Series 1 n=45/80 (56%)	Series 2-8 n=111/267 (42%)
One chromosomal region amplified (n=100; 29%)	**7p** **12q** **4q** **1q** 12p 7q 13q 6p 8q 15q 16q 17q	**17 (21%)** **3 (4%)** **4 (5%)** **2 (3%)** (0) (0) (0) (0) (0) (0) 1(1.2%) (0)	**46(17%)** **10 (4%)** **6 (2%)** 1 (0.4%) 2 (0.8%) 2 (0.8%) 2 (0.8%) 1 (0.4%) 1 (0.4%) 1 (0.4%) (0) 1 (0.4%)
Subtotal		**27 (34%)**	**73 (27%)**
Two chromosomal regions amplified (n=39; 11%)	**7p,12q** **1q,7p** **1q,4q** 7p,7q 3q,7p 4q,7p 4q,12q 1q,5p 1p7q 2q,12q 4q,9p 5p,7p 5q,7p 7p,11p 7p,9p 7p,14q 7p, Xp 17q,20q 20p,20q	**4 (5%)** **2 (3%)** (0) 1 (1.2%) (0) (0) 2 (3%) (0) 1 (1.2%) (0) (0) (0) (0) 1 (1.2%) (0) (0) (0) (0) (0)	**4 (1%)** **3 (1%)** **3 (1%)** 2 (0.8%) 2 (0.8%) 2 (0.8%) 2 (0.8%) 1 (0.4%) (0) 1 (0.4%) 1 (0.4%) 1 (0.4%) 1 (0.4%) (0) 1 (0.4%) 1 (0.4%) 1 (0.4%) 1 (0.4%) 1 (0.4%)
Subtotal		**11 (14%)**	**28 (10%)**
≥Three chromosomal regions amplified (n=17; 5%)	1p,1q,10q 1p,7p,12q 1p,12q,17q 1q,7p,12q 4p,4q,12p 4q,7p,12q 4q,7q,12p 4q7q12q 4q,7p,18q 5q,6q,7p 6p,7q,12q 7p,17p,17q 2p,7p,12p,12q 4q,7p,7q,15q 7p11p11q12q 7q,12q,17q,20q 1p,4p,7p,11q,14q	(0) (0) 1 (1.2%) 1 (1.2%) (0) 1 (1.2%) (0) 1 (1.2%) (0) 1 (1.2%) (0) 1 (1.2%) (0) (0) 1 (1.2%) (0) (0)	1 (0.4%) 1 (0.4%) (0) (0) 1 (0.4%) (0) 1 (0.4%) (0) 1 (0.4%) (0) 1 (0.4%) (0) 1 (0.4%) 1 (0.4%) (0) 1 (0.4%) 1 (0.4%)
Subtotal		**7 (9%)**	**10 (4%)**

Results expressed as number (percentage) of cases showing gene amplification/chromosome arm.

Tumors with isolated amplification of DNA sequences at a single chromosomal region had a median of 9 genes involved (range: 1 to 40 genes). Gene amplification at 7p11.2 included the smallest number of affected genes (median: 5 vs 16 genes for gene amplification involving DNA sequences at chromosomes 12q, 1q and 4q). In turn, cases showing amplified DNA sequences at several chromosomal regions had a median of 19 genes involved (range: 1 to 45 genes). The most commonly (>5% of cases) amplified genes per chromosomal region included: i) 9 genes at chromosome 1q32.1 (*SOX13, ETNK2, REN, KISS1, GOLT1A, PLEKHA6, PIK3C2B, MDM4* and *LRRN2)*; ii) 4 genes at chromosome 4q12 *(SCFD2, FIP1L1*, *PDGFRA* and *KIT*); iii) 5 genes in chromosome 7p11.2 *(EGFR, LANCL2, VSTM2A*, *VOPP1* and *SEC61G),* and; iv) multiple genes at chromosome 12 which had 3 cytobands involved, i.e. 12q14.1 (*CDK4, METTL1, CYP27B1, AVIL, CTDSP2, METT21B, AGAP2, OS9, TSFM*), 12q13.3 (*B4GALNT1, KIF5A, PIP4K2C, DTX3, SLC26A10, MARS, DCTN2, ARHGEF25*) and 12q15 (*ATP23, MDM2, CPM*) (Table 3). When individually considered, the *EGFR* gene (38%) together with the *LANCL2* gene (23%), coded also at chromosome 7p11.2, were the two most frequently amplified genes (Table 3). Of note, amplification of none of the genes detected here had been previously described in healthy individuals [33] and/or publicly available GBM databases.

**Table 3:** Frequency and chromosomal localization of recurrently amplified genes (> 5% of cases) in GBM from series 1 (n=45/80 tumors), and other previously reported series of GBM (n=111/267 tumors) as detected by SNP-arrays

Amplified genes					
Amplified chromosomal regions			Symbol	Frequency of gene amplification	
Chr	Amplified/Total cases (%)	Cytoband		Series 1 (n=45)	Series 2-8 (n=111)
Chr 7	119/347 (34%)	7p11.2	*EGFR* *LANCL2* *VSTM2A* *VOPP1* *SEC61G*	67% 40% 31% 27% 11%	57% 19% 29% 7% 19%
		12q14.1	*CDK4* *CYP27B1* *METTL1* *AVIL* *CTDSP2* *METT21B* *AGAP2* *OS9* *TSFM*	29% 29% 27% 27% 27% 22% 22% 16% 16%	14%^*^ 2%^*^ 14%^*^ 18%^*^ 2%^*^ 16%^*^ 16%^*^ 2%^*^ -
Chr 12	34/347 (10%)	12q13.3	*B4GALNT1* *KIF5A* *PIP4K2C* *SLC26A10^#^* *DTX3* *MARS* *ARHGEF25* *DCTN2^#^*	16% 11% 11% 11% 9% 9% 9% 7%	14%^*^ 10%^*^ - - 12%^*^ 4%^*^ 4%^*^ -
		12q15	*ATP23* *MDM2* *CPM*	16% 9% 9%	14%^*^ 12% 11%
Chr 4	26/347 (7%)	4q12	*SCFD2* *FIP1L1* *PDGFRA* KIT	18% 18% 18% 7%	7% 14% 10% 10%
Chr 1	14/347 (4%)	1q32.1	*SOX13* *ETNK2* *REN* *KISS1* *GOLT1A* *PLEKHA6* *PIK3C2B* *MDM4* *LRRN2*	11% 11% 11% 11% 11% 11% 11% 11% 9%	5% 3% 2% 3% 3% 5% 5% 3% 2%

#genes included in the SNP6 array; ^*^The incidence of amplified genes localized in the 12q14.1, 12q13.3 and 12q15 cytobands was calculated using only the 250k-SNP_Nsp and/or STY arrays (n=50) due to the absence of probes for these specific genes in the 50k array.

### Classification of GBM tumors based on their gene amplification profiles (GAP)

**Figure 2:**
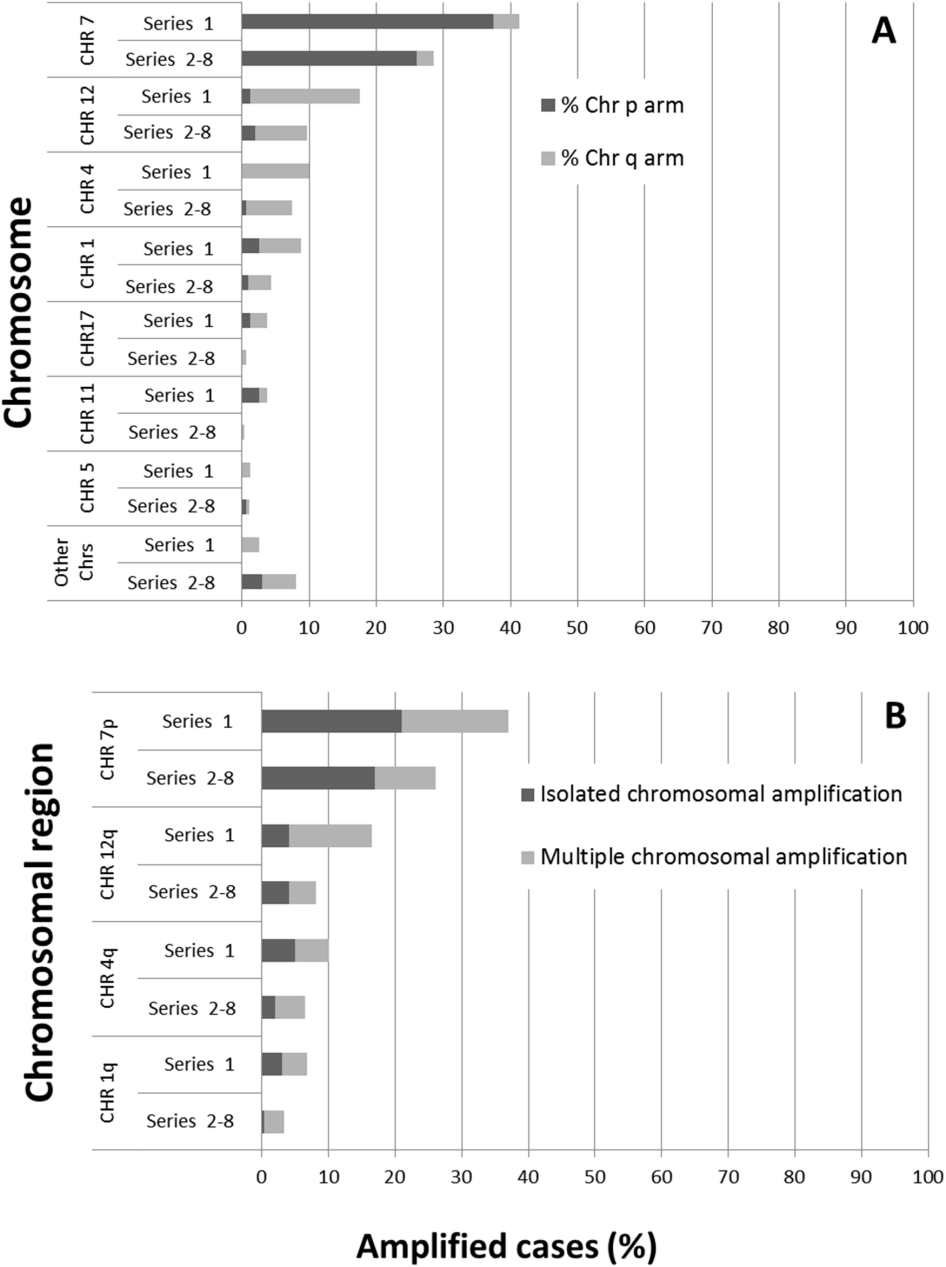
Distribution of the most frequently altered chromosomes **(A)** and chromosomal regions **(B)** showing isolated or multiple amplifications both in cases from series 1 (n=45/80) and in patients (111/267) from the seven distinct series previously reported in the literature by others. Results are shown as percentage values from all cases analyzed.

Based on the presence of gene amplification, their specific subtype and the number of chromosomal regions affected by gene amplification (Figure 1), GBM tumors from group 1 (series 1) were divided into five different subgroups: i) tumors which had no gene amplification (n=35; 44%); ii) tumors with isolated amplification of DNA sequences at chromosome 7p including the *EGFR* gene (n=17; 21%); iii) GBM with isolated amplification of DNA sequences at a chromosomal region different from chromosome 7p (n=10; 13%); iv) tumors with amplifications of DNA sequences from multiple (≥2) chromosomal regions including that of the *EGFR* gene (n=13; 16%), and; v) tumors with amplification of DNA sequences at ≥2 chromosomal regions which did not include amplification of the *EGFR* gene (n=5; 6%) (Table 4 and Figure 2).

**Table 4:** Overall survival and genetic features of GBM patients from our series (series 1; n=80) and the seven series of GBM patients previously reported in the literature (series 2-8; n=267) and included in this study for a total of 347 GBM investigated

Variables		GBM patient series							
		Series 1 Crespo et al.^*^ (GSE 42631)	Series 2 Chen et al. (GSE 19612)	Series 3 Beroukhim et al. (GSE19399/ GSE9635)	Series 4 Bralten et al. NA	Series 5 Hodgson et al. (GSE 14804)	Series 6 Yin et al. (EMEXP-1330)	Series 7 Kuga et al. (GSE 10922)	Series 8 Solomon et al. (GSE 13021)
Total N. of cases (n=347)		80	24	120	15	12	53	13	30
N. of cases with annotated OS (n=273)		80	24	104	15	NA	50	NA	NA
Median OS months (range)		15 (0-83)	15 (1-31)	18 (1-67)	10 (4-28)	NA	17 (0-90)	NA	NA
N. of SNP probes investigated		5-18×10^5^	5×10^5^	1-2.5×10^5^	2.5×10^5^	0.5×10^5^	0.5-2.5×10^5^	0.5×10^5^	2.5×10^5^
New genetical subsets	NO gene AMP (n=191; 55%)	35 (44%)	11 (46%)	75 (62%)	9 (60%)	7 (58%)	31 (58%)	7 (54%)	16 (53%)
	Isolated *EGFR* AMP (n=63; 18%)	17 (21%)	7 (29%)	20 (17%)	1 (7%)	2 (17%)	10 (19%)	2 (15%)	4 (13%)
	Isolated non-*EGFR* AMP (n=37; 11%)	10 (13%)	1 (4%)	11 (9%)	3 (20%)	2 (17%)	7 (13%)	1 (8%)	2 (7%)
	Multiple AMP including *EGFR* (n=36; 10%)	13 (16%)	3 (13%)	8 (7%)	1 (7%)	0	3 (6%)	2 (15%)	6 (20%)
	Multiple AMP without *EGFR* (n=20; 6%)	5 (6%)	2 (8%)	6 (5%)	1 (7%)	1 (8%)	2 (4%)	1 (8%)	2 (7%)

^*^23 additional GBM tumors not available in the GEO data repository were hybridized with the Cytoscan750K (n=11 samples) and Cytoscan HD (n=12 samples) SNP-arrays; AMP: genetic amplification; GAP: genetic amplification profile; GSE: genomic repository series code; OS: overall survival; N: number; NA: not available.

The above GAP were further investigated in the other 7 series of GBM used for validation purposes, for a total of 267 primary GBM patients (Table 4). Around half of these cases ˗ 156/267 (58%) ˗ did not show genetic amplification at any of the chromosomal regions investigated (Table 4); in contrast, 69 (26%) tumors had gene amplification at chromosome 7p11.2 amplification –46/267 cases (17%) with isolated *EGFR* amplification and 23/267 cases (9%) showed amplification of DNA sequences at multiple chromosomal regions including amplification of the *EGFR* gene– (Table 2 and Table 4). Similarly to what was observed in the test series, gene amplification at 7p, in association with gene amplification at chromosome 12q, was found in 6 tumors (2%) and gene amplification at chromosome 7p together with amplification of DNA sequences at chromosomal regions other than 12q was found in another 17 cases (6%), –e.g. gene amplification at the 1q or 4q chromosomal regions in 3/267 (1.1%) and 4/267 (1.5%) cases, respectively, and at 7q or 3q in 3/267 (1.1%) and 2/267 (0.8%) tumors, respectively – (Table 2). DNA sequences from other chromosomal regions which were also found to be amplified at lower frequencies than those amplified at 7p, included DNA sequences at chromosomes 12q (10/267; 4%) and 4q (6/267; 2%) (Table 2). Genetic amplification at other chromosomal regions such as 7q, 12p and 13q was found in two tumors each, and gene amplification at the 6p, 8q, 15q and 17q chromosomal regions was found in a single tumor each (Table 2).

Upon merging the test and validation series (series 1 to 8), the following distribution into the 5 subgroups of GBM defined by their distinct GAP (Table 4), was found: 191 cases (55%) had no gene amplification (group 1), 63/347 cases (18%) showed isolated amplification of *EGFR*) (group 2); 37/347 (11%) displayed isolated amplification of genes other than *EGFR* (group 3); 36/347 (10%) had genetic amplification at multiple chromosomal regions, including amplification of the *EGFR* gene (group 4); and 20/347 (6%) showed multiple amplified DNA sequences at ≥ 2 distinct chromosomal regions, which did not include amplification of the *EGFR* gene (group 5) (Table 4).

### Prognostic impact of gene amplification profiles in GBM

**Figure 3:**
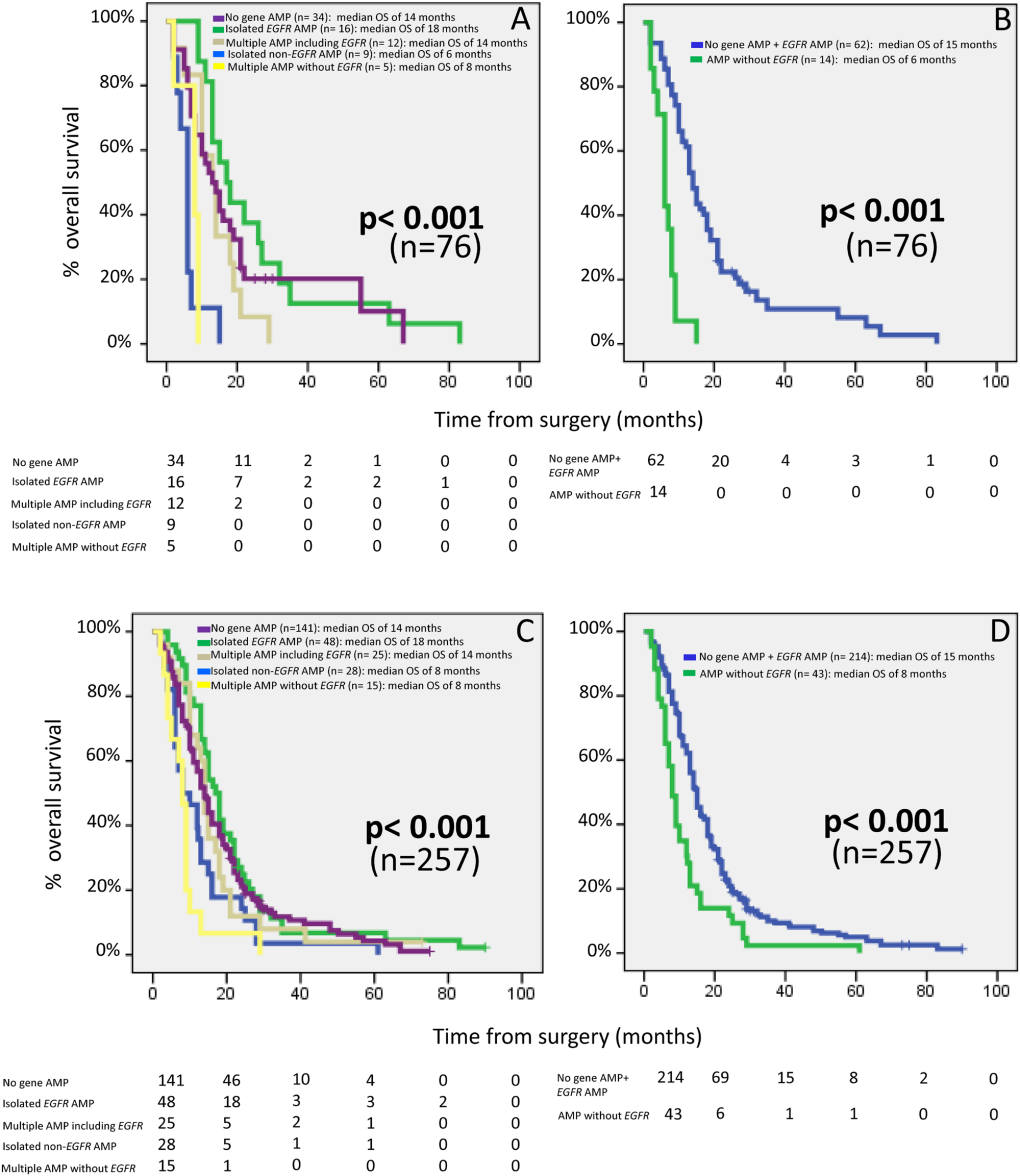
Prognostic impact on overall patient survival of distinct gene amplification profiles of GBM patients from the test cohort – series 1 alone (n=76); **panels (A)** and **(B)** – and after pooling our cases with 181 additional cases from seven series of GBM patients previously reported in the literature by other groups (**panels (C)** and **(D)**; n= 257 GBM). In panels (A) and (C), GBM patients were grouped as having i) no gene amplification; ii) isolated *EGFR* amplification; iii) gene amplification at multiple chromosomal regions including *EGFR* gene amplification; iv) isolated amplification of DNA sequences from a single chromosomal region other than 7p11.2 (i.e not including amplification of the *EGFR* gene), and; v) gene amplification at multiple chromosomal regions in the absence of *EGFR* amplification. In panels (B) and (D), patients were grouped as: i) cases showing either no gene amplification or having *EGFR* gene amplification and ii) patients showing genetic amplification at one or more chromosomal regions which did not involve the *EGFR* gene. Median overall survival is expressed in months and was calculated for 257 patients from series 1-8. Cases who were alive but had a follow-up of less than 18 months and/or died within the 1st month after surgery (n= 16) were excluded from OS analyses.

From the prognostic point of view, the above defined GAP showed a significant impact on OS of GBM patients, both in the test series (series 1) alone (p<0.001; Figure 3A), and in the whole cohort (series 1-8; p<0.001) (Figure 3C). In detail, cases that did not show gene amplification or that displayed amplification of the *EGFR* gene (alone or in combination with amplification of genes in other chromosomal regions) (groups 1, 2, and 4, respectively) showed a significantly better outcome than patients with isolated amplification of genes other than the *EGFR* gene (group 3) and cases with amplification of multiple chromosomal regions which did not involve the *EGFR* region (group 5): median OS of 14, 18 and 14 months vs 6 (p=0.001, p<0.001 and p=0.007, respectively) and 8 months (p=0.03, p<0.001 and p=0.003, respectively) in the test series alone, and of 14, 18 and 14 months vs 8 (p=0.03, p=0.003 and p=0.16, respectively) and 8 (p<0.001, p<0.001 and p=0.001, respectively) months in the whole cohort, respectively (Figure 3, panels A and C, respectively). When cases were re-grouped according to i) the absence of gene amplification or presence of *EGFR* gene amplification vs ii) occurrence of other GAP, the prognostic impact of the re-grouped GAP was enhanced, both when the test series alone and the whole cohort of patients analyzed, were considered: median OS rates of 15 vs 6 months (p< 0.001) and of 15 vs 8 months (p<0.001), respectively (Figure 3 panels B and D, respectively). Subsequent multivariate analysis of prognostic factors showed that the tumor GAP (p<0.001), together with the administration of chemotherapy (p<0.001) were the best combination of independent prognostic factors to predict patient OS (Table 5).

**Table 5:** Clinical characteristics of the GBM patients included in the series 1 (n=76) and their association to disease outcome

Clinical/genetic characteristics		Patient distribution	Overall survival			
			Univariate analysis		Multivariate analysis	
			Median (range)	*p-value*	Hazard Ratio (95% CI)	*p-value*
Age	>30	2 (2%)	67 (2.7-67)	<.001		
	31-45	9 (12%)	15 (8-21)			
	46-60	21 (28%)	14 (2-63)			
	61-75	34 (45%)	13 (2-83)			
	>75	10 (13%)	6 (2-11)			
Karnofsky Index ^#^	>70	48 (64%)	15 (2-83)	.004		
	≤70	27 (36%)	10 (2-30)			
Type of Surgery	Complete resection	24 (32%)	15 (2-83)	.012		
	Partial resection	41 (54%)	13 (2-67)			
	No resection	11 (14%)	6 (2-21)			
Adjuvant chemotherapy^#^	Stupp	43 (67%)	18 (2-83)	<.001	3 (2-4)	<.001
	BCNU	10 (16%)	13 (5-67)			
	No chemotherapy	11 (17%)	6 (2-11)			
Gene amplification profile	No gene AMP	34 (45%)	13 (2-67)	<.001		
	Isolated *EGFR* AMP	16 (21%)	17 (9-83)			
	Multiple AMP including *EGFR*	12 (16%)	13 (2-29)			
	Isolated non-*EGFR* AMP	9 (12%)	6 (2-15)			
	Multiple AMP without *EGFR*	5 (6%)	8 (2-9)			
	No gene AMP+*EGFR* AMP	62 (82%)	14 (2-83)	<.001	6 (2-12)	<.001
	AMP without *EGFR*	14 (18%)	6 (2-15)			

CI: confidence interval; ^#^: Data of Karnofsky index and adjuvant chemotherapy were only available in only 75 and 65 GBM patients respectively; Stupp: radiotherapy plus temozolamide; BCNU: carmustine; AMP: genetic amplification.

## DISCUSSION

Despite histopathology remains the gold standard for the diagnosis of GBM, it provides limited information about patient outcome. Consequently, new classifications have been proposed in recent years for GBM in which molecular genetics data derived from chromosomal alteration profiles, DNA mutational status and GEP data, is used for the definition of tumor subgroups of distinct embryonic tissue origins [13, 14]. However, these classification models are difficult to implement in routine diagnostics and/or remain of relatively limited prognostic value [34–36].

Among other cytogenetic/molecular alterations, gene amplification, particularly gene amplification involving the *EGFR* gene, represents one of the most common genetic changes in GBM [11, 20–22, 37]. Thus, *EGFR* amplification at chromosome 7p11.2 can be found in between one third and half of all GBM patients as the only chromosomal region amplified – including a variable number of amplified genes – or it can be find in the same tumor in combination with amplification of genes located at other chromosomal regions, such as the *MDM2, MDM4, PDGFRA* and *CDK4* genes encoded in chromosomes 12q15, 1q32.1, 4q12 and 12q14.1 [11, 14, 38], respectively. Of note, gene amplification is a common genetic alteration across different malignancies and it usually involves (wild-type or mutated) genes that show oncogenic potential (i.e are capable of reproducing the tumor); thereby, it might confer a malignant phenotype associated with a variable outcome, depending on the specific genes amplified and/or overexpressed. Despite this, no study has been reported so far in which the impact of different GAP on OS has been investigated in a large series of GBM patients.

Here we investigated the GAP across the whole GBM tumor genome and analyzed their impact on patient OS, based on one of the largest series of GBM patients reported so far in the literature. SNP-arrays were used for both sensitive identification of CNA involving specific DNA sequences across the whole tumor genome and detailed delineation of the amplified genes; in order to avoid CNV due to germinal single nucleotide polymorphisms, insertions and deletions potentially associated with an increased predisposition to GBM (e.g. the rs1801320 SNP in the RAD51 DNA repair gene [39]), paired tumor and peripheral blood (PB) samples were analyzed per patient.

Overall, our results showed the presence of gene amplification in the majority (>50%) of tumors investigated. As expected, genetic amplification involving DNA sequences at the 7p11.2 chromosomal region was by far the most frequent alteration, followed by genetic amplification at the 12q, 4q12 and 1q32.1 chromosomal regions, and to a lower extent also, at 1q, 5q, 6q, 7q and at regions in both arms of chromosomes 11 or 17. These results confirm and extend on previous observations by our [20–22] and other groups [40, 41] which indicate that *EGFR* is the most frequently amplified oncogene in GBM, where it is detected in up to 40% of primary GBM tumors in association with a better outcome, compared to cases that show either no gene amplification or amplification of genes other than *EGFR* [20, 37]. At present, it is well-established that activation of the *EGFR* gene via gene amplification and/or mutations, up-regulates the RAS/RAF/MAPK and PI3K signaling pathways, translating into a tumor phenotype consisting of: i) abnormally high cell proliferation and ii) survival of tumor cells, and iii) an increased angiogenesis [11, 42]. Of note, here we confirm via mapping of the amplified region in chromosome 7p11.2, that this alteration frequently includes also the *LANCL2* gene, in addition to other genes adjacent to the EGFR and *LANCL2* genes [20, 43]. Although the *LANCL2* gene codes for a protein involved in up-regulation of AKT and cell survival, and an increased cell sensitivity to adriamycin [44, 45], its precise mechanism of action remains elusive.

In line with previous observations [11, 38, 46], *EGFR* gene amplification at the 7p11.2 chromosomal region, was found either as the only amplified DNA sequence, or in association with amplification of DNA sequences at other chromosomal regions and genes such as the *PDGFRA, MDM2, MDM4* and *CDK4* genes. Such combined pattern of amplification of multiple genes at distinct chromosomal regions might lead to unique malignant transformation profiles for which the underlying mechanisms are still poorly understood; however, in our series, it did not prove to confer a more adverse outcome vs isolated amp 7p11.2. In contrast, isolated amplification of DNA sequences at chromosomal regions other than that containing the *EGFR* gene, were associated with a significantly shorter OS of GBM patients. Of note, these later alterations most frequently affected genes encoded in the 12q, 4q and 1q chromosomal regions, and they typically involved multiple genes [15]. Thus, amplification of DNA sequences at the 12q13-14 chromosomal region usually included the *CDK4* gene together with the *METTL1, CYP27B1, AVIL, CTDSP2, METT21B, AGAP2* and *OS9* genes, while genetic amplification at 12q15 affected the *MDM2* oncogene in a significant fraction of all (primary) GBM tumors analyzed. *CDK4* is a member of the Ser/Thr protein kinase family, required for the cell cycle transition from the G1 to S-phase [47]; thus, *CDK4* phosphorylates the *Rb* gene product leading to its inactivation and the release of proteins required for cell cycle progression, at the same time it also down-regulates *TP53* [47]. In turn, *AVIL* binds actin and promotes the development of neuronal cells, while the *MDM2* gene codes for a nuclear-localized E3-ubiquitin ligase whose transcription is recognized as the main p53 negative regulator [48]. In our series, genetic amplification at the 4q11-12 chromosomal region, systematically affected the *PDGFRA* gene together with the *SCFD2* and *FIP1L1* genes. *PDFGRA* [11] codes for a tirosine-protein kinase cell surface receptor of the PDGF growth factor, which promotes cell proliferation and migration. Finally, amplification of DNA sequences at chromosome 1q32.1 involved the *MDM4* gene together with the *SOX13, ETNK2, KISS1, GOLT1A, PLEKHA6, REN* and *PIK3C2B* genes. MDM4 inhibits *TP53* and *TP73,* mediating cell cycle arrest via binding to their transcriptional activation domain, at the same time it inhibits degradation of *MDM2* [48], whereas *PIK3C2B* belongs to the PI3K gene family and activates signaling for cell proliferation, survival and migration [49]. In turn, the *DTX3* ubiquitin ligase gene probably acts both as a positive and negative regulator of Notch, depending on the developmental stage and cell context [50].

Altogether, these findings indicate that the distinct GAP here reported for GBM tumors might confer a distinct biological and (also) clinical behavior to these tumors. Thus, based on the presence vs absence of gene amplification and its subtypes, five different patterns were defined among our GBM patients, which showed an association with OS. From the prognostic point of view, these five GAP could be further re-grouped into two major risk-groups including: i) patients with either no gene amplification or *EGFR* gene amplification associated with or without amplification of genes coded at chromosomal regions other than chromosome 7p11.2, with a significantly longer OS; and, ii) cases presenting with amplification of one or more chromosomal regions that did not include *EGFR* gene amplification, and that were associated with a significantly poorer outcome. The prognostic impact of this later classification was further confirmed in a larger cohort of GBM patients previously reported in the literature and, together with the type of treatment administered, emerged as the most powerful combination of independent prognostic factor for GBM patients. However, the precise mechanisms involved in determining the distinct survival rates of these two molecular groups of patients, still remain to be elucidated and deserve further investigations.

Despite several classification models have been previously proposed which address the genomic heterogeneity of GBM [13, 14, 27] and identify tumors with different cellular origins, so far they have proven to be of limited prognostic value [34–36, 51] and/or difficult to be used in routine diagnostics due to the complexity of the information they require to classify GBM patients at diagnosis. In contrast, here we propose a relatively simple prognostic stratification model for GBM tumors based on their gene amplification profiles that might be easily implemented in routine laboratory diagnostics, and that will potentially contribute to a better management of the patients. In line with this, we have recently patented an array containing this combination of probes [52] to assess the above referred GAP, and that we hope can be commercially available for routine diagnostics soon.

## MATERIALS AND METHODS

### Patients and samples

Overall, 347 GBM tumors were studied. These included two groups of adult patients: the first group consisted of 80 caucasian GBM patients (group 1, series 1) with histological diagnosis of primary GBM based on the WHO criteria (38 males and 42 females; mean age of 62±13 years, ranging from 24 to 84 years) (Table 6). Fifty-seven of these 80 patients (71%) were admitted to the University Hospital of Coimbra (Coimbra, Portugal) and their genomic data has been deposited in the genomic repository GEO (series code number: GSE42631) and 23 (29%) were from the University Hospital of Salamanca (Spain). Each patient from series 1 gave his/her informed consent prior to entering the study, and the study was approved by the local Ethics Committees of both institutions, according to the Declaration of Helsinki. For each patient within this first group, tumor samples containing representative areas of (fresh) tumor tissues were obtained by surgical resection, immediately (<30 min) snap-frozen in liquid nitrogen, and stored at -80°C for further SNP-array studies; in parallel, a PB sample was also collected from each patient. Prior to the SNP-array studies, a section was cut from the stored tissue blocks and assessed by conventional histopathological procedures for its tumoral cell contents. Specimens with ≥75% tumoral cells, in the absence of significant contamination by normal brain parenchyma and tumoral necrosis, were selected for further DNA and RNA extraction.

**Table 6:** Clinical and biological characteristics of GBM patients from series 1 who were analyzed by single-nucleotide polymorphism arrays in this study (n=80)

Case ID	Age	Gender	Karnofsky Index (%)	Location	Brain hemisphere	Overall survival or follow-up	Exitus	Treatment	
								Type of surgery	Chemotherapy
GBM1	80	M	ND	Temporal	L	6	Yes	T	ND
GBM2	75	M	80	Fronto-temporal	R	22	Yes	T	Stupp
GBM3	61	F	90	Parietal	R	83	Yes	T	Stupp
GBM4	73	M	100	Temporal	L	19	Yes	P	Stupp
GBM5	38	M	90	Frontal	R	15	Yes	T	Stupp
GBM6	49	F	60	Frontal	R	30^*^	No	P	Stupp
GBM7	41	M	40	Temporal	R	11	Yes	P	Stupp
GBM8	57	M	90	Tempo-parietal	R	6	Yes	T	Stupp
GBM9	72	M	90	Temporal	R	28	No	P	Stupp
GBM10	62	M	100	Parietal	R	28	No	T	Stupp
GBM11	71	M	80	Temporal	R	27	Yes	P	Stupp
GBM12	50	F	100	Temporal	R	13	Yes	T	Stupp
GBM13	72	F	70	Temporal	R	9	Yes	P	Stupp
GBM14	78	F	100	Frontal	R	6	Yes	T	-
GBM15	61	F	100	Frontal	R	25	No	T	Stupp
GBM16	54	M	100	Fronto-parietal	R	2	Yes	T	Stupp
GBM17	52	M	80	Frontal	R	63	Yes	P	Stupp
GBM18	57	F	90	Temporal	R	10	Yes	P	Stupp
GBM19	68	M	90	Occipital	L	10	Yes	T	Stupp
GBM20	82	F	80	Frontal	L	7	Yes	T	-
GBM21	77	M	70	Temporal	R	6	Yes	P	-
GBM22	69	F	100	Frontal	L	8	Yes	T	Stupp
GBM23	24	F	80	Frontal	L	21	Yes	P	Stupp
G97	53	M	80	Temporal	R	21	Yes	T	Stupp
G94	79	F	80	Temporal	R	9	Yes	P	-
G93	63	M	80	Occipital	R	29	Yes	T	Stupp
G92	54	F	80	Parietal	R	15	Yes	T	Stupp
G91	73	F	60	Occipital	R	13	Yes	P	Stupp
G90	57	F	60	Parietal	L	5	Yes	B	-
G89	51	M	80	Temporal	R	2	Yes	P	-
G88	71	M	80	Parietal	R	8	Yes	P	Stupp
G87	45	M	80	Temporal	L	16	Yes	P	Stupp/ Sequential
G83	75	M	70	Temporal	R	10	Yes	P	-
G82	78	M	70	Frontal	R	2	Yes	B	-
G81	62	F	70	Frontal	R	13	Yes	P	Stupp
G80	43	M	80	Frontal	R	18	Yes	T	Stupp
G79	71	F	60	Occipital	R	6	Yes	B	-
G73	78	F	60	Parietal	L	4	Yes	B	-
G72	77	F	70	Temporal	L	1	Yes	P	-
G71	66	F	60	Parietal	R	10	Yes	P	Sequential
G70	56	F	80	Occipital	L	21	Yes	P	Stupp
G68	72	M	70	Insular	L	26	Yes	T	Stupp
G67	68	F	80	Parietal	R	35	Yes	P	Stupp
G66	60	M	80	Occipital	R	14	Yes	T	Stupp
G65	69	F	60	Parietal	L	1	Yes	P	-
G64	57	M	60	Occipital	L	8	Yes	P	Sequential
G63	61	F	60	Insular	R	13	Yes	P	Sequential
G62	57	F	90	Occipital	R	18	Yes	T	Stupp
G57	34	M	90	Frontal	R	8	Yes	T	Stupp
G56	65	M	80	Frontal	L	13	Yes	P	Stupp
G55	54	F	80	Frontal	R	17	Yes	P	Stupp
G54	65	F	60	Parietal	L	6	Yes	P	-
G53	74	M	60	Frontal	L	29	Yes	T	Stupp
G52	56	M	90	Frontal	L	21	Yes	B	Stupp
G51	60	M	60	Temporal	R	2	Yes	B	-
G50	84	M	70	Temporal	R	11	Yes	P	-
G46	62	M	60	Frontal	L	3	Yes	P	-
G45	76	F	60	Temporal	R	10	Yes	P	-
G44	48	M	80	Frontal	L	22	Yes	P	PCV
G43	67	F	70	Temporal	R	7	Yes	P	-
G42	67	M	80	Temporal	R	2	Yes	P	-
G41	44	F	60	Frontal	R	14	Yes	B	Sequential
G40	45	F	80	Frontal	R	15	Yes	P	BCNU+TMZ
G39	70	F	50	Frontal	R	18	Yes	P	Stupp
G37	70	M	80	Temporal	R	32	Yes	T	Stupp
G35	50	F	80	Frontal	L	2	Yes	P	-
G34	69	M	60	Temporal	R	5	Yes	B	-
G31	71	F	90	Frontal	R	7	Yes	P	-
G30	71	F	70	Temporal	R	9	Yes	B	-
G29	49	F	80	Parietal	L	12	Yes	B	Sequential
G25	68	M	80	Frontal	L	6	Yes	P	Stupp
G23	50	F	70	Frontal	R	14	Yes	B	Stupp
G17	30	F	90	Temporal	R	67	Yes	P	Sequential
G15	79	M	80	Parietal	L	5	Yes	T	Sequential
G14	69	F	70	Frontal	R	0	Yes	B	-
G13	39	F	90	Frontal	R	20	Yes	P	Sequential
G12	74	M	70	Temporal	R	1	Yes	B	-
G10	35	F	80	Temporal	L	15	Yes	P	Stupp
G8	67	F	90	Deep	NA	9	Yes	P	Stupp
G6	70	F	80	Temporal	R	19	Yes	P	Stupp

The second group of GBM patients included 267 unselected cases from 7 different series of GBM (series 2 to 8; group 2) previously reported in the literature [15, 24, 28–32]. Data from cases included in one of these series (series 4; n=15 cases) were kindly provided by Bralten et al [32], while data about the patients and tumor samples from the other six series was accessed from publicly available data bases – GSE19612 (series 2; 24 cases); GSE19399 and GSE9635 (series 3; 104 and 16 cases, respectively); GSE14804 (series 5; 12 cases); E-MEXP1330 (series 6, 53 cases); GSE10922 (series 7; 13 cases) and GSE13021 (series 8; 30 cases)–.

### DNA extraction and identification of copy number alterations by SNP-arrays

DNA from frozen tumor samples (n=80 tumors from series 1) was purified using the QIAamp DNA Mini Kit (Qiagen, Valencia, CA, USA), according to the instructions of the manufacturer. The yield and purity of the extracted DNA were determined using a NanoDrop-1000 spectrophotometer (Nano-Drop Technologies Inc, Wilmington, DE, USA), and they systematically showed absorbance (A) values >1.5 at 260/230nm and ≥1.8 at 260/280nm wavelengths, respectively. DNA integrity was evaluated by conventional electrophoretic procedures in a 1% agarose gel. RNA extraction and cDNA synthesis were performed following the standard operating procedures (SOPs) of the Spanish DNA Bank Carlos III (University of Salamanca, Salamanca, Spain) (http://www.bancoadn.org).

For the investigation of CNAs by SNP-arrays, DNA from frozen tumor tissues and their paired PB samples was used in order to exclude individual CNV due to germline SNPs, small insertions and deletions. Briefly, extracted DNA (250ng per array) was digested with restriction enzymes and ligated to the corresponding adaptors, following conventional Affymetrix procedures (Affymetrix Inc, Thermo-Fisher Scientific, Waltham, MA, USA). A generic primer that recognizes the adaptor sequence was used in triplicate, to amplify adaptor-ligated DNA fragments via polymerase chain reaction (PCR). The amplified DNA was then fragmented, labeled, and hybridized to the corresponding SNP-array (please see below). After hybridization, chips were washed in an Affymetrix Fluidics Station 450 (Affymetrix) and the hybridized sequences were labeled using streptavidin-phycoerythrin, and assayed by fluorescence detection using a GeneChip Scanner 3000 (Affymetrix). The allelotype at a locus was then determined based on probe-associated fluorescence intensity data for oligonucleotides complementary to the reference sequences that covered the corresponding SNP position.

In the test series (series 1), 4 different types of SNP-arrays were used. These included: i) the GeneChip Human Mapping 500K Array Set (n=35 tumors), which provides information about >500,000 SNPs according to the NCBI/hg19 assembly (262,264 SNPs in the Nsp array and 238,304 SNPs in the Sty array); ii) the Genome-Wide Human SNP Array 6.0 (n=22 cases), which contains probes for 906,600 SNPs and 945,826 non-polymorphic probes featuring a total of >1.8 million probes (Affymetrix); and, iii) the CytoScan 750K and Cytoscan HD arrays (n=23 tumors) which contain probes for 200,436 SNPs and 743,304 non-polymorphic probes (Affymetrix). Data about DNA probes was analyzed with the Console Genotyping software (version 3.0.2; Affymetrix). In addition, the dChip 2010 software (http://www.dchip.org; Dana Farber Cancer Institute, Boston, MA, USA) was used to calculate CNA values. To plot CNAs according to their chromosomal location, the Chromosome Analysis Suite (CHAS) was used. The Hg 19 human genome sequence was used as reference to name the amplified genes, as defined by CNAs values > 4.8 (arbitrary units) typically corresponding to > 7 DNA copies (in order to exclude polyploidies) [29].

For the validation series, data on 7 different SNP-array chips (Affymetrix 50K, 100K, 250K and/or 500K SNP-arrays) previously reported by others [15, 23, 24, 28–32] were used for the analysis of CNA. The number of common SNPs for the 100K and 500k arrays, and for the 500K and cytoscan HD arrays, was of 21,144 and 65,535 SNPs, respectively, for a total of around 11,000 SNPs in common to all SNPs-arrays used.

### Mutational analyses

Analysis of *IDH1* and *IDH2* gene mutations was based on DNA extracted from formalin-fixed and paraffin-embebed tissues (n=54) using the QIAamp DNA Mini kit (Qiagen, Germany) according to the instructions of the manufacture. Exon 4 DNA of both the *IDH1* and the *IDH2* genes was amplified by PCR and sequenced on a capillary automated sequencer (CEQ 8000; Beckman-Coulter, Hialeah, FL, USA); mutational analysis of the sequence data was performed using the Sequencher, (version 4.7) software (Genes Codes, Ann Arbor, MI, USA). None of the 54 primary GBM cases analyzed showed *IDH1* or *IDH2* mutations.

### Statistical analysis

The statistical significance of differences observed between groups was assessed by the Student T and the Mann-Whitney U tests, for parametric and non-parametric (continuous) variables, respectively; for categorical variables, the *X^2^* test was used. Overall, 257 GBM who survived for >1 month after surgery and had a minimum follow-up of 18 months (for patients remaining alive) were included in OS analyses. Survival curves were plotted according to the method of Kaplan and Meier, and the (two-sided) log-rank test was used to assess the statistical significance of differences in OS among distinct groups of patients. Multivariate analysis of prognostic factors for OS was performed using the Cox stepwise regression model. In this part of the study, only those variables showing a significant association with RFS in the univariate analysis were included (Table 5). *P*-values <0.05 were considered to be associated with statistical significance. For all statistical analysis, the SPSS software (SPSS 17.0, IBM SPSS, Armonk, NY, USA), was used.
